# 

**Published:** 1915-05-29

**Authors:** 


### Buildings Contemplated and in Progress.

Brandon.—The Urban District Council have decided to forward to the Local Government Board plans and estimates for the new infectious diseases hospital.

Bury.—The new isolation block at the Bury Infirmary has been formally opened. Mr. H. C. Cooper, of Bury, was the architect.

Chadderton.—The Lancashire County Council have decided to apply for sanction to borrow £11,500 for erecting a smallpox hospital at Chadderton.

Colne.—The management committee of the Colne Cottage Hospital have under consideration an offer made by Sir William Hartley to erect and equip a new hospital with the most modern appliances and outfit for twenty-four beds, and to present it to the town on condition that the present hospital buildings be adapted for use as a convalescent home in connection with the new hospital.

Dewsbury.—Tenders are invited for the erection of certain buildings in connection with the Whitley Sanatorium. Quantities may be obtained of the Borough Surveyor, Town Hall, Dewsbury.

Ely.—Plans have been passed for an isolation hospital at Ely, on a site at Mill Pits. The cost of the building is estimated at £1,000.

Glamorgan.—At a meeting of the Glamorganshire Joint Poor-Law Establishment Committee at Neath, it was decided to proceed with the negotiations with the trustees of the Drymma and Brithdir estates for the purchase of the estates as sites for a home for the feeble-minded. The architect has been directed to prepare plans for an institution for 700 patients.

Great Barr.—The Health Committee recommend the Walsall Town Council to purchase, in conjunction with the West Bromwich Town Council, about thirty acres at Barr Common, Great Barr, as a site for a sanatorium. The scheme involves an expenditure of £20,000, to be borne jointly by Walsall and West Bromwich.

Hexham.—With regard to the proposal of the Rural District Council to build a new hospital, the Local Government Board have asked to be furnished with complete information and plans.

Leek.—At the last meeting of the Rural District Council, Mr. W. H. Walley, architect, Stockton Brook, presented plans of a new hospital. The estimated cost is £3,100, for a building of brick with stone foundations, or £3,176 for a building all of stone. The plans were referred to the hospital committee for consideration.

Lisnaskea.—The Guardians propose to carry out alterations in the wards of the fever hospital with a view to providing accommodation for the nurses.

Llechryd.—An isolation hospital is about to be built at Llechryd to serve the districts of Rhymney and Tredegar.

Rates of Subscription : Twelve months, 6/6 ; Six months, 4/- ; Three months, 2/-, post free to any address in the United Kingdom. Abroad, Twelve months, 8/8; Six months, 5/-; Three months, 2/6, post free.—Address The Subscription Dept., "The Hospital," The Hospital Building, 28/29 Southampton Street, Strand, London, W.C.

*Now Ready.* Twenty-Sixth Year of Publication. Over 1,000 pages. In Scarlet Cloth.

Price 10/6 net. By post, 10/11.

BURDETT'S

HOSPITALS and CHARITIES, 1915.

The Year Book of Philanthropy and Hospital Annual.

Edited by Sir HENRY BURDETT, K.C.B., K.C.V.O.

"The Ideal of what a work of reference ought to be."—*The Times.*

The volume for 1915 is the 26th Issue of "Hospitals and Charities," and contains SPECIALLY WRITTEN CHAPTERS on :—

The Volume of Charity, 1913. The Future of the Hospitals. The King's Fund and the League of Mercy. The Nursing Department in 1913 and its Cost. Hospital Sunday. Hospital Saturday. Missions. Orphanages. The Blind. The Deaf and Dumb. Hospital Finance—Income, 1913 ; Expenditure, 1913. The United States, Canada, Australasia, and India. Convalescent Institutions. Hospital Construction, 1914.

ALL WITH CAREFULLY ANALYSED FIGURES, AND A DIRECTORY OF

Medical Universities, Colleges, and Schools.—London Hospitals.—London Dispensaries.—Metropolitan Asylums Board Hospitals.—Poor-Law Infirmaries in London and the Provinces.—Provincial Hospitals.—Provincial Infectious Hospitals.—Provincial Dispensaries.—Scotch Hospitals.—Scotch Dispensaries.—Irish Hospitals.—Lunacy Board and Lunatic Asylums : Indian, British, and Colonial.—Military and Naval Hospitals.—Convalescent Homes.—Sanatoria for Consumption.—Retreats for Inebriates.—Private Asylums.—Nursing Institutions.—Indian, Colonial, and American Hospitals.—Hospitals for Insane in the United States of America.—Administrative and Collective Societies.—Missionary and Religious Societies.—Orphanages, Homes, and Charities.—Institutions for the Blind.—Institutions for the Deaf and Dumb.—London County Council Centres for Blind Children.—London and Provincial Institutions for the Deaf and Dumb.—London County Council Centres for Deaf and Dumb Children.—Hospitals and Homes for Incurables.—Establishments for the Relief of Epilepsy.—Vaccine Lymph Establishments.—Reformatory and Industrial Schools.

Obtainable of all Booksellers. Publishers : The Scientific Press, Ltd., London.

